# Investigation on the Tool Wear Suppression Mechanism in Non-Resonant Vibration-Assisted Micro Milling

**DOI:** 10.3390/mi11040380

**Published:** 2020-04-03

**Authors:** Lu Zheng, Wanqun Chen, Dehong Huo

**Affiliations:** 1 Mechanical Engineering, School of Engineering, Newcastle University, Newcastle upon Tyne NE1 7RU, UK; L.Zheng5@newcastle.ac.uk; 2 Centre for Precision Engineering, Harbin Institute of Technology, Harbin 150001, China; chwq@hit.edu.cn

**Keywords:** vibration-assisted machining, micro milling, tool wear, tool-workpiece separation

## Abstract

Excessive tool wear during hard and brittle material processing severely influences cutting performance. As one of the advanced machining technologies, vibration-assisted micro milling adds high-frequency small amplitude vibration on a micro milling tool or workpiece to improve cutting performance, especially for hard and brittle materials. In this paper, the tool wear suppression mechanism in non-resonant vibration-assisted micro milling is studied by using both finite element simulation and experiment methods. A finite element model of vibration-assisted micro milling using ABAQUS is developed based on the Johnson cook material and damage models. The tool-workpiece separation conditions are studied by considering the tool tip trajectories. The machining experiments are carried out on Ti-6Al-4V with a coated micro milling tool (fine-grain tungsten carbide substrate with ZrO2-BaCrO4 (ZB) coating) under different vibration frequencies (high, medium, and low) and cutting states (tool-workpiece separation or non-separation). The results show that tool wear can be reduced effectively in vibration-assisted micro milling due to different wear suppression mechanisms. The relationship between tool wear and cutting performance is studied, and the results indicate that besides tool wear reduction, better surface finish, lower burrs, and smaller chips can also be obtained as vibration assistance is added.

## 1. Introduction

Many industrial sections have been striving towards product miniaturization. In order to obtain superior physical, mechanical, optical, and electronic properties, hard and brittle materials, such as titanium alloy, silicon carbide ceramic, and optical glass, have been chosen for micro products, e.g., medical devices, bio-sensors, micro-fluidic chips, to name a few [1,2]. As one of the most efficient micro-manufacturing methods, micro-end milling has shown a great capacity in micro product processing and can generate complex geometries on a wide range of materials. Due to the well-known size effect in micro milling, cutting edge radius can be no longer ignored, which leads a different cutting mechanism to its conventional scale counterpart [3]. The machining performance can be influenced by the tool edge radius when the uncut chip thickness is small enough. It has been reported that the ratio of uncut chip thickness to cutting edge radius is closely linked with the machining surface ploughing effect, effective rake angle, and specific cutting energy, which in turn impacts the cutting performance [4,5,6]. Moreover, when it refers to hard and brittle materials, low fracture toughness and often poor thermal conductivity make processing these materials a challenging task. A series of defects, such as high tool wear rate and fracture damage, will occur during the machining process, which leads to a low machined surface quality and machining accuracy [7,8,9]. Although many efforts, including cutting tool geometry and machining parameter optimization, enhance the processing performance, it still cannot meet the increasing demands on high productivity precision micro machining.

As an unconventional machining method, vibration-assisted micro milling is a cost effective way to process hard and brittle materials by imposing high frequency small amplitude vibration to the cutting tool or workpieces [10,11]. Compared with the conventional machining process, several advantages can be obtained, such as burr suppression, lower cutting force, better surface quality, higher machining accuracy, and functional surface texture generation [12,13,14,15,16,17]. In addition, it also shows great potential for tool wear reduction. Ding et al. [18] studied the cutting performance in two-dimensional vibration-assisted micro-end-milling on steel experimentally and found the surface finish and cutting tool life to be improved. Chandra et al. [19] developed an ultrasonic vibration-assisted machining system and investigated the influence of vibration and machining parameters on cutting tool wear. The experiment results indicate that the cutting tool life can increase 4–8 times as vibration is added. Li et al. [20] investigated the vibration-assisted micro milling process and found that the tool life is extended compared with conventional milling results and better surface roughness and lower burrs can be obtained because it reduces the secondary damage of the worn cutting tool to the machined surface effectively. Zhang et al. [21] studied diamond tools’ wear process in the vibration-assisted machining of steel. By comparing the results of workpiece temperature and cutting energy consumption between vibration-assisted machining and conventional machining, they validated the notion that temperature reduction is not the main reason for diamond tool wear suppression in a vibration-assisted machining process and raised their own conjecture: gas pressure increased at the cutting interface and machined surface oxide layer generation. Javad et al. [22] studied the relationship between cutting speed and tool life in the ultrasonic milling process and found that tool wear is only reduced under specific parameters. However, none of these studies have a systematic description of the mechanism of tool wear suppression in vibration-assisted machining, particularly for precision micro milling.

In this paper, the tool-workpiece separation conditions are studied first to obtain the appropriate vibration and cutting parameters. The tool wear suppression mechanism in vibration-assisted micro milling is studied through analysing both machining simulation and experiment results. The results show that the tool wear can be reduced effectively when the tool-workpiece separation occurs, as well as a higher applied vibration frequency. In addition, better surface finish, lower burrs, and smaller chips can also be found when vibration assistance is added. 

## 2. Analysis of Tool-Workpiece Separation 

### 2.1. Tool Tip Trajectory 

The tool-workpiece separation in the vibration-assisted micro milling process is determined by both the vibration and machining parameters and plays an important role in the tool wear. In order to study the tool-workpieces separation conditions, the tool tip trajectories in the conventional milling process need to be understood first and its motion equations can be expressed as:

(1) $$\{\begin{array}{c} X_{i}=V_{f}t+R\sin\left(\frac{n\pi t}{30}+i\pi \right)\\ Y_{i}=R\cos\left(\frac{n\pi t}{30}+i\pi \right) \end{array}$$

where *X_i_* and *Y_i_* represent the coordinates of the tool tip positions in *X* and *Y* directions, respectively. *X* is used as the feed direction and *Y* is used as the cross-feed direction in this research. *i* indexes the cutting edges (two flute end mills are used in this research, so *i* = 0 or 1), *V_f_* (mm/s) is the cutting speed, *n* (rev/min) is the spindle speed, *R* (mm) is the cutting tool radius, and *t* (s) is the cutting time. 

When a sinusoidal signal vibration is added to the feed direction, the relative trajectories between the tool tips and the workpiece can be expressed as:

(2) $$\{\begin{array}{c} X_{i}=V_{f}t+R\sin\left(\frac{n\pi t}{30}+i\pi \right)+A_{x}\sin\left(2\pi f_{x}t+\varphi_{x}\right)\\ Y_{i}=R\cos\left(\frac{n\pi t}{30}+i\pi \right) \end{array}\;\;\;\;\;\;\;\;\;\;\;\;\;\;\;\;\;\;\;\;\;\;\;\;$$

where *f_x_* (Hz) and *A_x_* (µm) are the vibration frequency and amplitude, respectively, and $\varphi_{x}$ is the initial vibration phase angle.

### 2.2. Tool-Workpiece Separation Conditions

**Figure 1:**
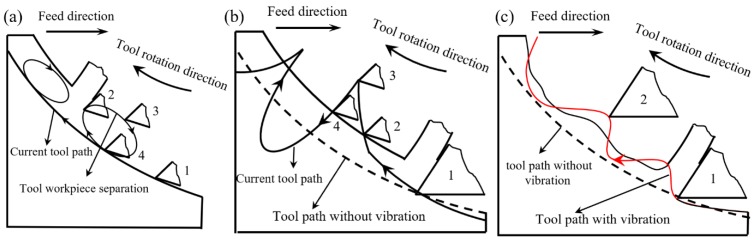
Three types of tool-workpiece separation conditions. (**a**)Type I; (**b**) Type II; (**c**) Type III.

According to the tool tip trajectory analysis, three different continuous tool-workpiece separation conditions can be obtained in the vibration-assisted milling process. Figure 1a shows the first-type separation state, which appears when the relative speed between the tool and workpiece in the cutting direction goes against the spindle speed direction. Tool motion can be expressed as the following four steps: In step 1, the cutting tool starts to contact the workpiece and the relative speed is positive. In step 2, the cutting tool is about to break contact with the workpiece and the relative speed is equal to zero. In step 3, the relative speed is reversed and the cutting tool withdraws from the workpiece. In step 4, the relative speed is positive again and another cutting cycle is started. 

Therefore, as the necessary condition for achieving the first type tool-workpiece separation, the tool or workpiece vibration speed (*V_v_*) needs to be greater than the nominal cutting speed (*V_n_*, the relative speed of the tool tip without vibration assistance), which can be expressed as:

(3) $$[V_{v}=2\pi f_{x}A_{x}\cos\left(2\pi f_{x}t+\varphi_{x}\right)cos\alpha ]\geq [V_{n}=\frac{\pi rn}{30}]$$

Simplify Equation (3)

(4) $$\cos\left(2\pi f_{x}t+\varphi_{x}\right)cos\alpha \geq \frac{rn}{60f_{x}A_{x}}$$

Equation (4) is only valid when:

(5) $$\frac{rn}{60f_{x}A_{x}}\leq 1$$

Figure 1b shows the second type separation state, it is determined by the relationship between the vibration amplitude and the instantaneous uncut chip thickness, and the separation process can also be expressed as the following four steps: In step 1, the cutting tool starts to contact the workpiece, and, at that time, the vibration amplitude is smaller than the instantaneous uncut chip thickness. In step 2, the instantaneous uncut chip thickness is zero and the cutting tool and workpiece are ready to separate. In step 3, the cutting tool loses contact with the workpiece and the value of vibration displacement is larger than the instantaneous uncut chip thickness. In step 4, the cutting tool recontact the workpiece and another cutting cycle is started.

**Figure 2:**
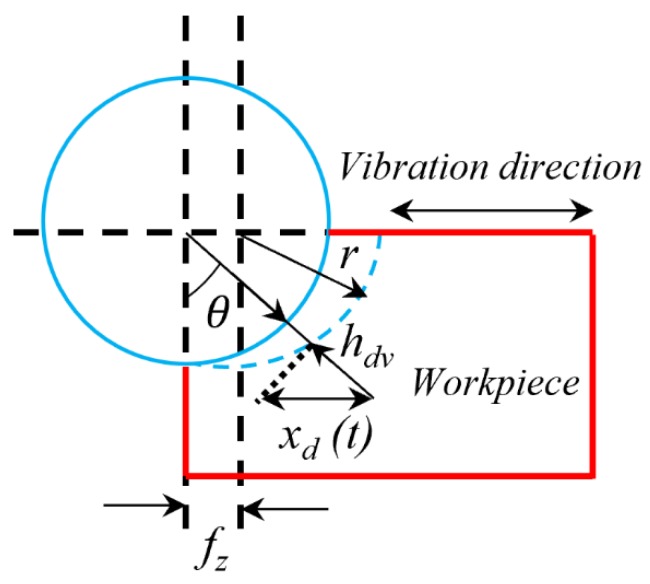
Schematic diagram of instantaneous uncut chip thickness.

Figure 2 shows the layout of instantaneous uncut chip thickness, and uncut chip thickness in vibration-assisted machining *h_dv_* can be expressed as:

(6) $$h_{dv}=f_{z}sin\theta +x_{d}sin\theta =\left(f_{z}+x_{d}\right)sin\theta$$

where *f_z_* is the feed rate, and the second separation occurs when $f_{z}+x_{d}$ < 0.

**Figure 3:**
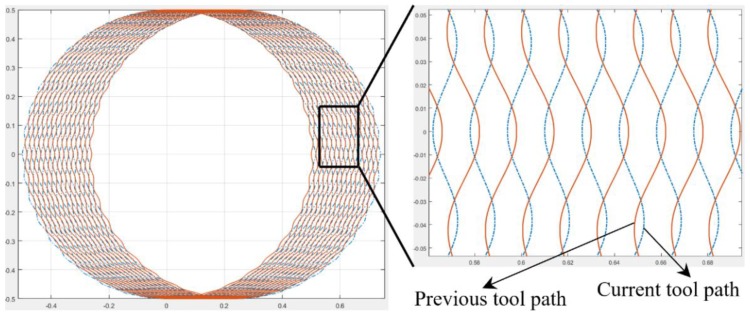
Tool trajectories for the third-type separation condition.

Figure 1c shows the third-type separation state by considering the interaction between the current and previous tool tip trajectories. Figure 3 shows the tool trajectories’ simulation results, and the trajectory interaction can be found between the current and previous tool trajectories. This happens when the applied vibration frequency consists of odd numbers of the spindle rotation frequency, and the vibration amplitude is greater than half of the feed per tooth. 

## 3. Finite Element Simulations

**Figure 4:**
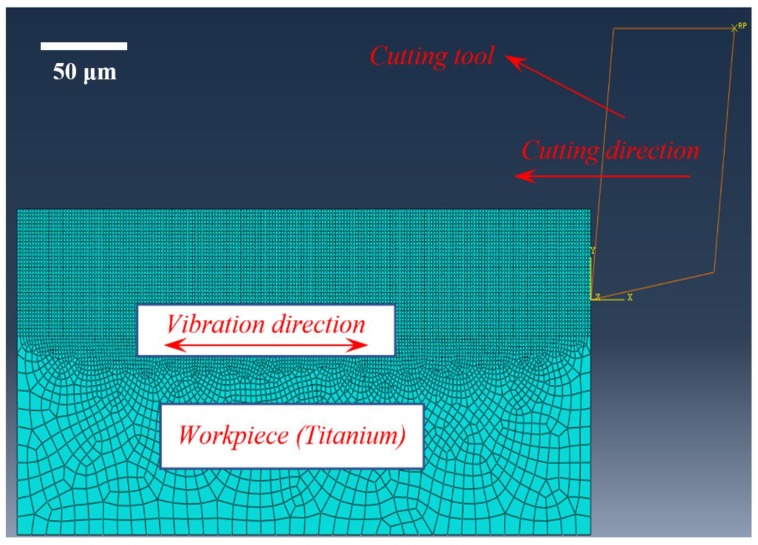
Finite element (FE) model of vibration-assisted machining.

Finite element simulations have been successfully utilized to investigate cutting mechanism over the past few decades. To further understand the relationships between tool wear and cutting performance, a finite element (FE) model is built using ABAQUS/Explicit commercial finite element software (Dassault Systèmes, Velizie Veracubray, France), as shown in Figure 4. Different vibrations are applied to the Ti-6Al-4V workpiece and the cutting tool was set as an analytical rigid body with a predefined cutting speed. The data of No. 1, 6 and 7 in Table 1 are selected as the vibration and machining parameters applied in the Finite element (FE) model. The workpiece material behaviour is described by the Johnson–Cook (J–C) model, and the material and damage models and the relevant parameters are shown in Table 2. The cutting process usually involves large deformation and a high deformation rate; therefore, the workpiece is meshed with a four-node quad-dominated element (CPE4R), localized encryption is performed in the large deformation area, the size of the mesh is decreased until no effect on the calculated results, and the average mesh size in this model is set at 5 μm. To avoid excessive mesh distortion in every analysis increment, arbitrary Lagrangian–Eulerian (ALE) formulation with the advancing front algorithm is selected. To describe the contact condition between the cutting tool and workpiece, the Coulomb friction model is used with a friction coefficient of 0.5 [23]. 

## 4. Experimental Setup 

**Figure 5:**
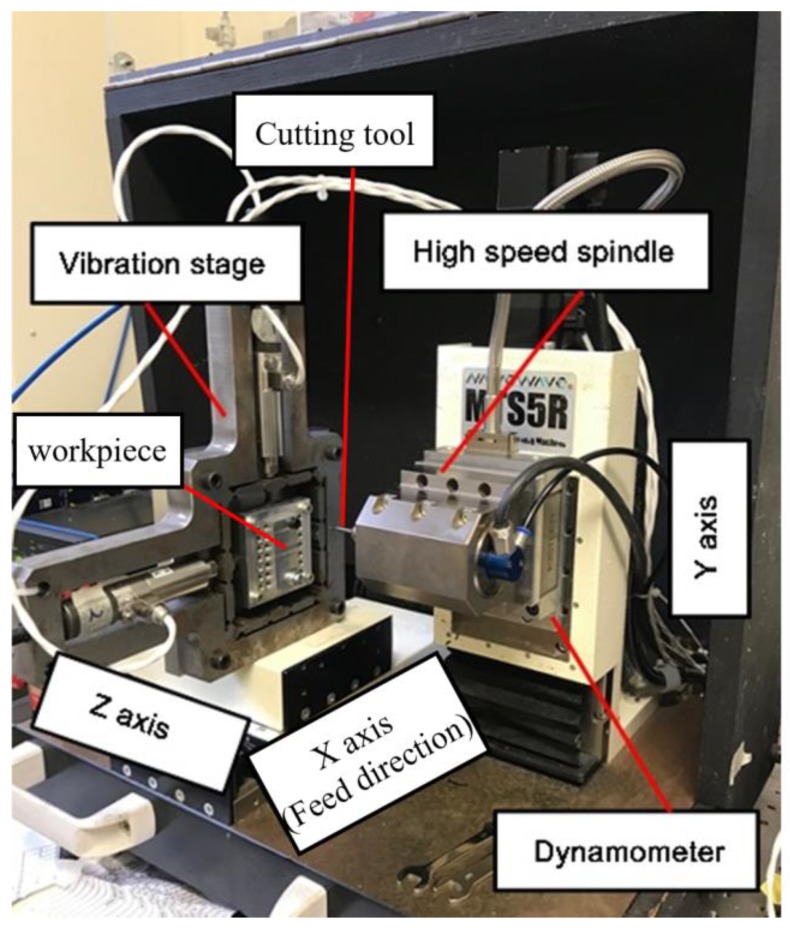
Layout of vibration-assisted micro milling equipment.

**Figure 6:**
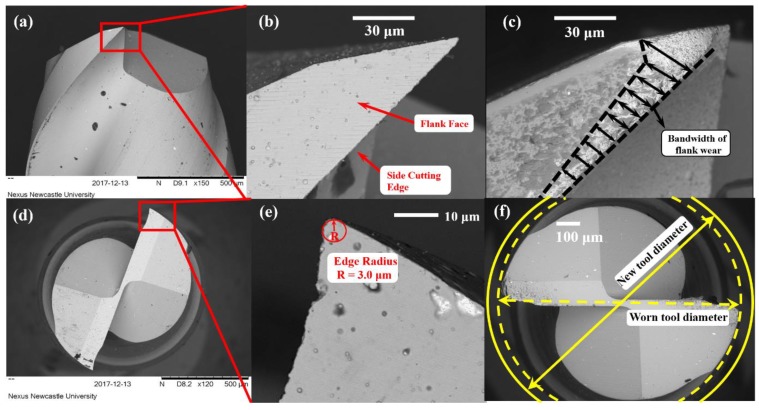
SEM results of cutting tools. (**a**) A side view of a brand new tool; (**b**) the flank face of the brand new tool; (**c**) the measurement method of the tool flank wear; (**d**) a top view of brand new tool; (**e**) the measurement method of the tool edge radius; (**f**) the measurement method of the worn tool diameter loss.

The machining experiments were conducted on a desktop precision micro-milling machine (Nanowave MTS5R, Nano Corporation, Yokohama, Japan) equipped with a high-speed spindle (max 80,000 rpm). Figure 5 shows the designed vibration stage mounted on the machine tool, and a dynamometer (Kistler 9256C1, Kistler Group, Winterthur, Switzerland) is set at the high-speed spindle side to measure cutting force. The slot milling experiments were carried out on Ti-6Al-4V using two 1-mm diameter flutes coated end mills with helix angles of 45°, whose substrates is the fine-grain tungsten carbides and coated with ZrO_2_-BaCrO_4_ (ZB). The geometry of the brand new micro end mills was checked using a scanning electron microscope (SEM) (TM3030, Hitachi, Tokyo, Japan), and their cutting edge radius was measured to be 3.0 μm in average (Figure 6e). In order to monitor the tool wear accurately, the cutting edge radius, flank wear, and tool effective diameter reduction, which greatly affect the processing performance, are selected as tool wear criteria. The cutting edge radius variation and tool effective diameter reduction were measured as shown in Figure 6e,f, respectively. Figure 6c shows the measurement method of tool flank wear (*VB*), which is explained as the band width in the direction perpendicular to the cutting edge. Moreover, in this paper, the average value of flank wear is chosen for describing tool flank wear by measuring ten different positions on the worn tool flank face (Figure 6c). Moreover, each result is measured 10 times to reduce the measurement error. All the micro cutters are selected from the same batch to eliminate the effects of tool manufacturing errors. 

The machining and vibration parameters are shown in Table 1 and only feed direction vibration is applied in these experiments. In order to reduce the influence of the ploughing effect on the whole experiments, the feed rate is set to 1.5 μm/tooth, which is larger than the minimum chip thickness (0.3–0.4 times of the cutting edge radius). The vibration parameters are selected according to the analysis results on the tool-workpiece separation conditions in Section 2.2. It aims to achieve the tool-workpiece separation and non-separation under three (high, medium and low) different frequencies by changing the vibration amplitudes. In addition, a conventional machining with the same machining parameters is carried out as a control experiment to analyse the effect of the vibration parameters on tool wear. The cutting tools are cleaned by the ultrasonic bath and checked by the SEM after every 100 mm cutting length. The machining results, such as burrs, surface roughness, and chips, are checked by the SEM and a white light interferometer (Zygo NewView 5200, Zygo Corporation, Middlefield, CT, USA), respectively.

## 5. Results and Discussion 

### 5.1. Tool Wear Results and Wear Mechanism 

**Figure 7:**
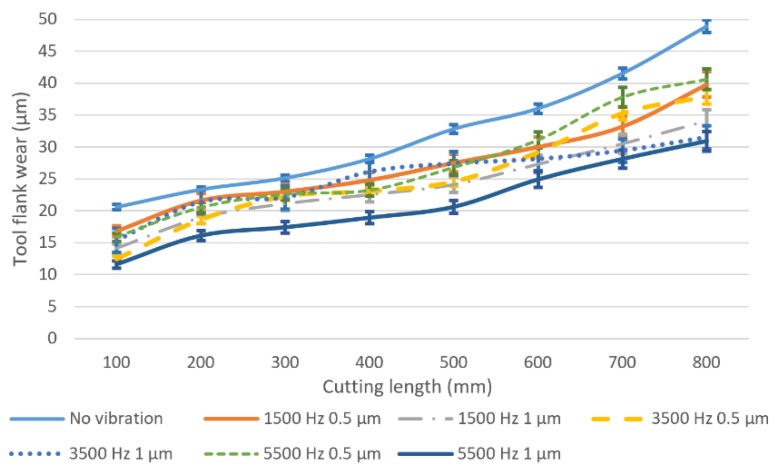
Tool flank wear under different vibration conditions and cutting length.

**Figure 8:**
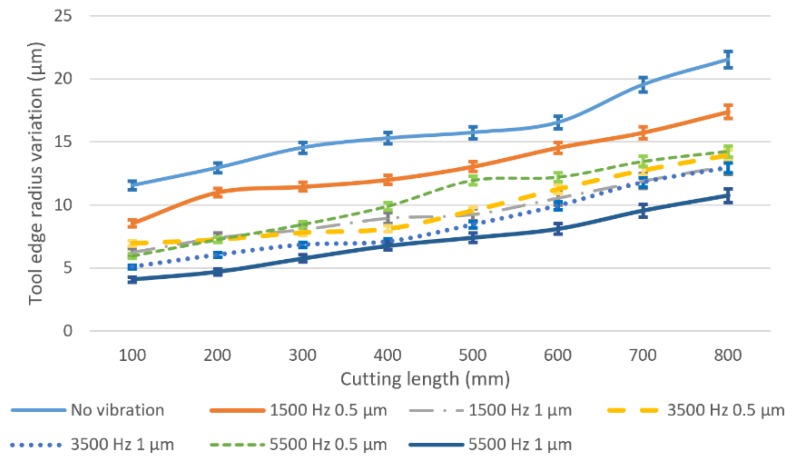
Tool edge radius variation under different vibration conditions and cutting length.

**Figure 9:**
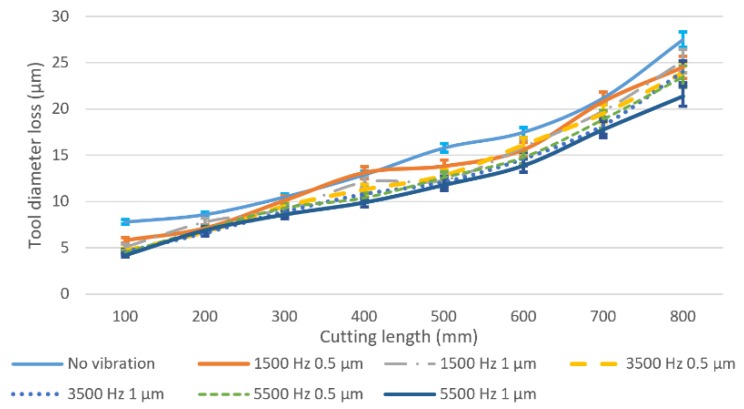
Tool diameter loss under different vibration conditions and cutting lengths.

The results obtained from the machining experiments have shown that the tool wear is significantly affected by the vibration conditions and the results for tool flank wear. The cutting edge radius variation, and tool effective diameter reduction are shown in Figure 7, Figure 8 and Figure 9, respectively. Figure 7 illustrates the results of the tool flank wear under different vibration conditions. The tool flank wear gradually increases with the increase in cutting length in all cutting experiments. Greater flank wear is observed in conventional micro milling at all cutting lengths, which means vibration assistance has a positive effect on reducing tool flank wear. When the vibration is added, the tool average flank wear length decreases by about 20% to 40% depending on the vibration conditions, and the minimal wear is recorded at the vibration frequency of 5500 Hz and an amplitude of 1 um. It can be observed that higher vibration frequency and tool-workpiece separation can effectively reduce the average tool flank wear. Generally, the tool wear behaviour can be divided into three stages. In the first wear stage, in this experiment, a cutting length from the start to approximately 200 mm and a relatively high tool wear rate can be observed. During this stage, the contact area is comparatively small due to the brand new tool sharp cutting edge, which leads to a high pressure and hence speeds up the tool wear. With the progression of tool wear, the contact area between tool flank face and workpiece on the worn tool cutting edge becomes lager and smoother. As a result, the pressure is reduced and the tool wear rate slows down. The tool wear enters the third stage as the cutting length reaches 600 mm in this experiment and a higher tool wear rate is observed on average tool flank wear on the results of both conventional micro milling and vibration-assisted micro milling without tool-workpiece separation. This may be caused by the increase in the tool edge radius (Figure 8) which exacerbates the influence of the size and ploughing effect and hence speeds up the tool wear. On the other hand, the rate of average tool flank wear in the condition of tool-workpiece separation is relatively stable beyond the cutting length of 600 mm. This can be attributed to the significant variation of instantaneous uncut chip thickness and cutting speed due to periodic contact between the tool and workpiece, which reduces the time of the squeeze friction between the tool and the workpiece as well as extends the cutting length of second stage. 

Figure 8 shows the results of the tool cutting edge radius variation for different vibration conditions. It can be found that, in conventional milling, the tool cutting edge radius increases dramatically and reaches almost 11.5 µm at 100 mm of cutting length due to the sharp edge of the brand new tools and high cutting forces. When vibration is applied, the rate of the cutting edge becoming blunt has decreased significantly. The lowest worn tool cutting edge radius can be observed at the vibration frequency of 5500 Hz and an amplitude of 1 µm. Generally, when keeping the amplitude constant, the increased rate of cutting edge radius will decrease as the vibration frequency decreases at the same cutting length. Higher vibration amplitude can suppress the cutting edge becoming blunt when the vibration frequency is constant, which agrees with the tool flank wear results. 

The accuracy of the milling tool diameter has a great influence on machining accuracy. Figure 9 shows the worn tool diameter loss under different vibration conditions. It can be observed that the tool diameter loss under all vibration conditions increases as the cutting length increases, and the cutting tool in conventional milling has the highest tool diameter loss from 8 µm at the cutting length of 100 mm to the 28 µm at the cutting length of 800 mm. When adding vibration to the milling process and fixing the vibration amplitude at 0.5 µm, a 25% to 40% tool diameter loss reduction can be obtained through changing vibration frequencies (1500 Hz, 3500 Hz and 5500 Hz) compared with the conventional milling results at the cutting length of 100 mm. A further reduction, 33% to 50% for the three different frequencies, can be reached when the vibration amplitude is fixed to 1 µm. Moreover, the tool diameter loss in the higher vibration amplitude (1 µm) is usually lower than that of the lower vibration amplitude at the same vibration frequency, which is contributed to by the lesser contact time of the tool with the workpiece, thus lowering the tool abrasive wear at the tool-workpiece separation condition. 

**Figure 10:**
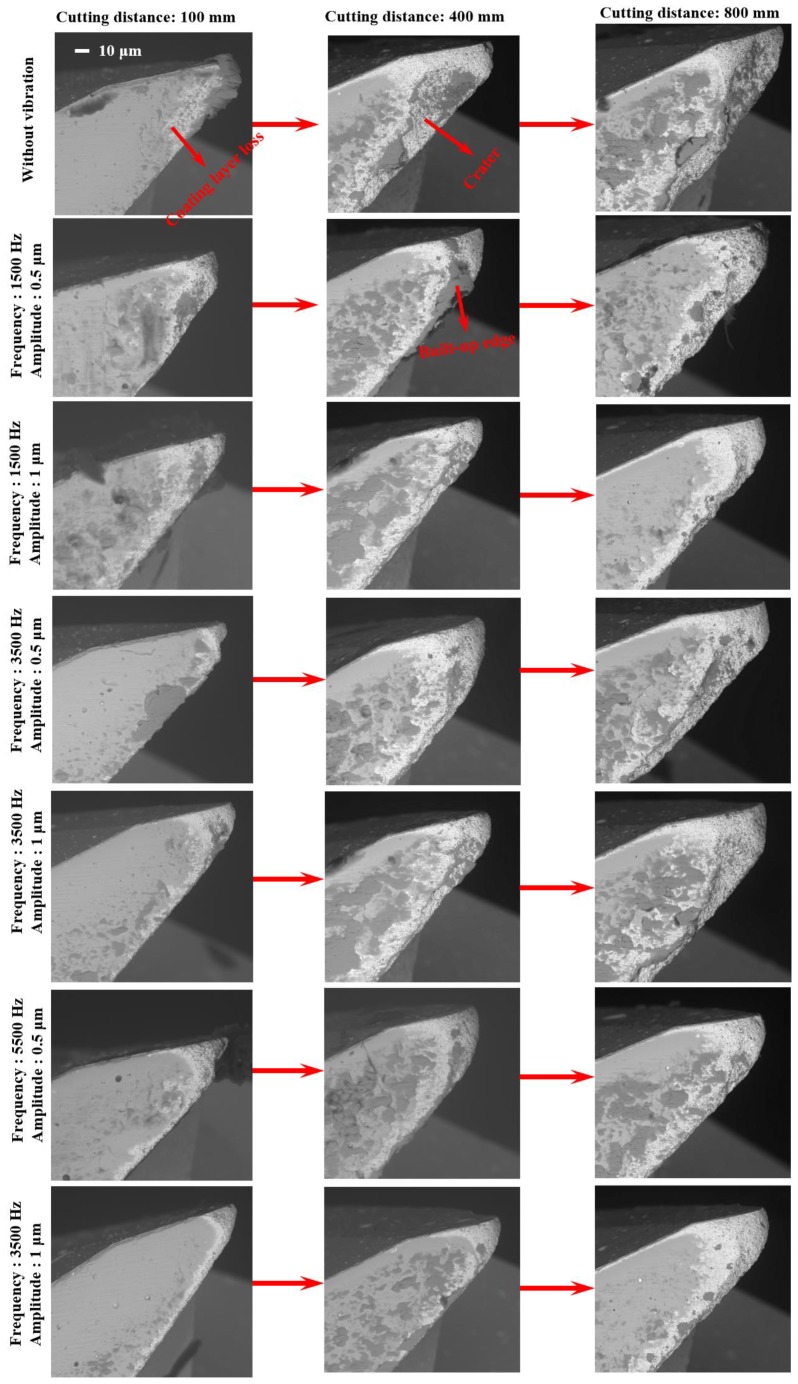
SEM results of flank face for the worn tools under different vibration conditions and different cutting lengths.

**Figure 11:**
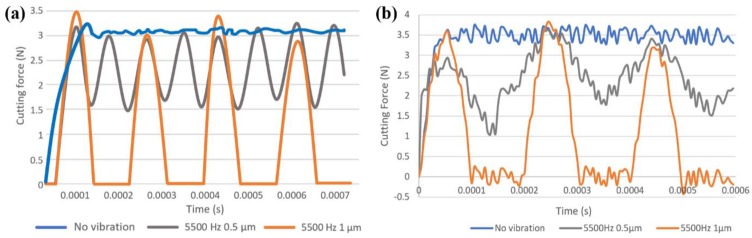
Results of cutting force, (**a**) the FE model, and (**b**) the machining test.

Figure 10 shows the SEM results of flank face for the worn tools under different vibration conditions. SEM images of tool wear at cutting lengths of 100, 400, and 800 mm are presented. It can be found that the tool wear varies with different vibration conditions at the same cutting length and the cutting tool in conventional milling is the most worn tools among these conditions. To further understand the reasons causing tool wear results differently in conventional micro milling and vibration-assisted micro milling, wear mechanisms are studied. In these wear mechanisms, coating layer loss (in Figure 10), which is reported to be caused by a chemical reaction or crack propagation caused by the difference in thermal expansion coefficient between the coating and the substrate, is believed to be the initial wear mechanism for coated tool [25]. As an important indicator of coating layer loss, the area of coating layer loss is highly related to the properties of the coating layer material and the value of the cutting force. And for the ZB coating, the friction coefficient will increase with temperature, increasing at relatively low temperatures [26]. According to the tool wear results in Figure 10, coating layer loss can be found in all experimental conditions. The cutting tool in conventional micro milling obtains the largest area of coating layer loss compared with that in vibration-assisted micro milling. In addition, in the proposed experiment conditions, heat generation in the cutting zone is limited due to the low axial depth of the cut, the feed rate, and the relatively short cutting length per SEM checking cycle. As vibration is added, lower cutting force, which is verified by both machining and FE results (Figure 11), and cutting temperature can be obtained, leading to a lower value for the friction coefficient of ZB coating and reducing the coating galling effect. It also can be found that the coating layer loss area in the condition of tool-workpiece separation is smaller than that in tool-workpiece non-separation under the same vibration frequency and the same cutting length. This can be contributed to by the periodic separation effect between the cutting tool and the workpiece, which not only promotes the cutting tool heat dissipation and reduces the cutting temperature, leading to a lower friction coefficient, but also further decreases the average cutting force due to the cyclical fluctuation of cutting forces (from 0 to almost 3.5 N), which is shown in Figure 11. As a result, a smaller area of coating layer loss can be obtained.

**Figure 12:**
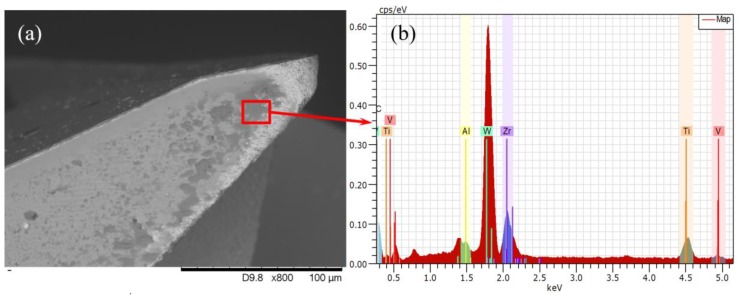
SEM image and energy dispersive spectrometer (EDS) spectra of worn tool. (**a**) SEM image; (**b**) EDS plots.

Adhesive wear is believed to be one of the dominant wear mechanisms of a coated carbide tool when processing titanium alloys, leading to cutting tool failure. It always happens as the coating layer loss takes place or is worn out completely. Large pressure, strong friction, and high temperature can speed up the adhesive wear progress. In the Figure 10, bonded workpiece materials can be observed at the flank face in all experimental conditions and the result of energy dispersive spectrometer (EDS) (TM3030, Hitachi, Japan) analysis (see Figure 12) reveals high concentrations of titanium, confirming that the adhesive wear takes place [27]. The results of the conventional machining are studied first to investigate the progress of adhesive wear in conventional micro milling. In the early wear stage, the bonded workpiece material at the cutting edge gradually increases with the cutting length and finally form a built-up edge, which can be found in Figure 10 of conventional micro milling at a cutting length of 100 mm. As the worn tool continues to impact and squash, the built-up edge begins to crack and eventually causes the substrate at the cutting edge breakage, forming craters, which can be found in Figure 10 of the conventional milling at a cutting length of 400 mm. This wear process repeats as the cutting length increases, causing the craters to become bigger and bigger until the cutting tool fails, which is shown in Figure 10 of a conventional milling at cutting length of 800 mm. As the vibration is added to the conventional micro milling process, the tool wear results in Figure 10 show that the adhesive wear progress is postponed backward. In the early wear stage (cutting length of 100 mm), a smaller built-up edge on the cutting tool can be found in the results of vibration-assisted micro milling. As the cutting length increases, the crater on the cutting tool of vibration-assisted micro milling is also smaller than that of the conventional micro milling. In addition, the adhesive wear in vibration-assisted micro milling also varies with the different vibration conditions. The amount of bonded workpiece material and the size of the craters on the worn cutting tool will be reduced when increasing the vibration frequency and satisfying the condition of tool-workpiece separation. Moreover, the best result can be obtained on the cutting tool results of the vibration condition 5500 Hz and 1 µm. This benefits from its smaller cutting forces, processing time reduction, and lower tool temperature. 

**Figure 13:**
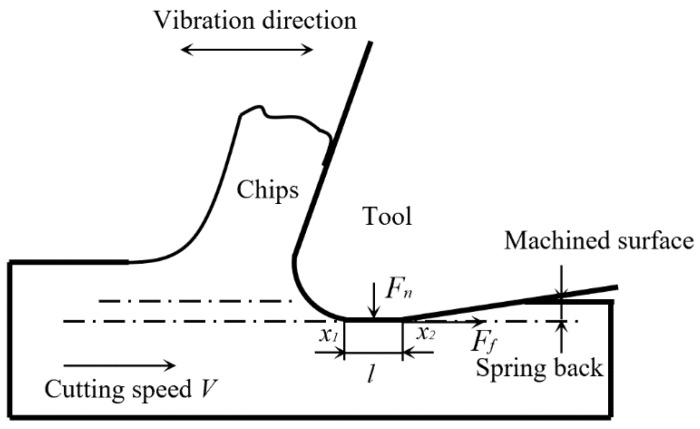
Layout of contact length between the workpiece and the tool in vibration-assisted micro milling.

Mechanical wear caused by the strong friction between the tool and workpiece is also one of the wear mechanisms in these experiments. The results also show that the mechanical wear can be reduced effectively under the conditions of a higher vibration frequency and tool-workpiece separation, which is again contributed to by the smaller friction force. Figure 13 shows the layout of the contact length between the workpiece and the tool in vibration-assisted micro milling, and high frequency reciprocating motion can be obtained by the cutting tool.

In Figure 13, the initial contact length between the tool flank face and workpiece is *l*. When vibration is added on the cutting tool, the actual contact length can be expressed as:

(7) $$\begin{array}{c} L=l+Asin2\pi ft \end{array}$$

where *t* is the cutting time and *f* and *A* are the vibration frequency and amplitude, respectively.

Therefore, the relative position of two contact points *x*_1_ and *x*_2_ can be expressed as:

(8) $$\begin{array}{c} x_{1}=Vt+\frac{L}{2}=Vt+\frac{l+Asin2\pi ft}{2} \end{array}$$

(9) $$\begin{array}{c} x_{2}=Vt-\frac{L}{2}=Vt-\frac{l+Asin2\pi ft}{2} \end{array}$$

where *V* is the cutting speed.

The relative speed of the two contact points, *V_1_* and *V_2_*, can be expressed as:

(10) $$V_{1}={\dot{x}}_{1}=V+\pi fAcos2\pi ft=V+V_{c}cos2\pi ft$$

(11) $$V_{2}={\dot{x}}_{2}=V-\pi fAcos2\pi ft=V-V_{c}cos2\pi ft$$

where *V_c_* = 2π*fA* is the critical velocity.

Then, the total friction force on the tool flank face *F_t_*(*t*) can be expressed as:

(12) $$\begin{array}{c} F_{t}\left(t\right)=\frac{\mu F_{n}}{2}\left[sgn\left(V+V_{c}cos2\pi ft\right)+sgn\left(V-V_{c}cos2\pi ft\right)\right] \end{array}$$

where *F_n_* is the fixed normal force between the two contact points, *µ* is the coefficient of friction.

And the average of the total friction force *F_a_* during a vibration cycle can be expressed as:

(13) $$\begin{array}{c} F_{a}=\frac{1}{T}\int_{0}^{T}F_{t}\left(t\right)dt=\{\begin{array}{ll} \frac{2\mu F_{n}}{\pi }\arcsin\left(\frac{V}{V_{c}}\right) & \left(V<V_{c}\right)\\ \mu F_{n} & \left(V\geq V_{c}\right) \end{array} \end{array}$$

According to Equation (13), the friction force on the tool flank face is *µF_n_* when cutting speed *V* is larger than or equal to the critical velocity *V_c_*. This process is similar to the normal machining process, so the direction of the friction *F_r_* does not change during the machining process. The friction force on the tool flank face is much less than *µF_n_* when cutting speed *V* is less than the critical velocity *V_c_*. Moreover, the direction of the friction changes periodically with the separation of the tool from the workpiece, which in turn reduces tool mechanical wear [28].

### 5.2. Relationship between Tool Wear and Cutting Performance

**Figure 14:**
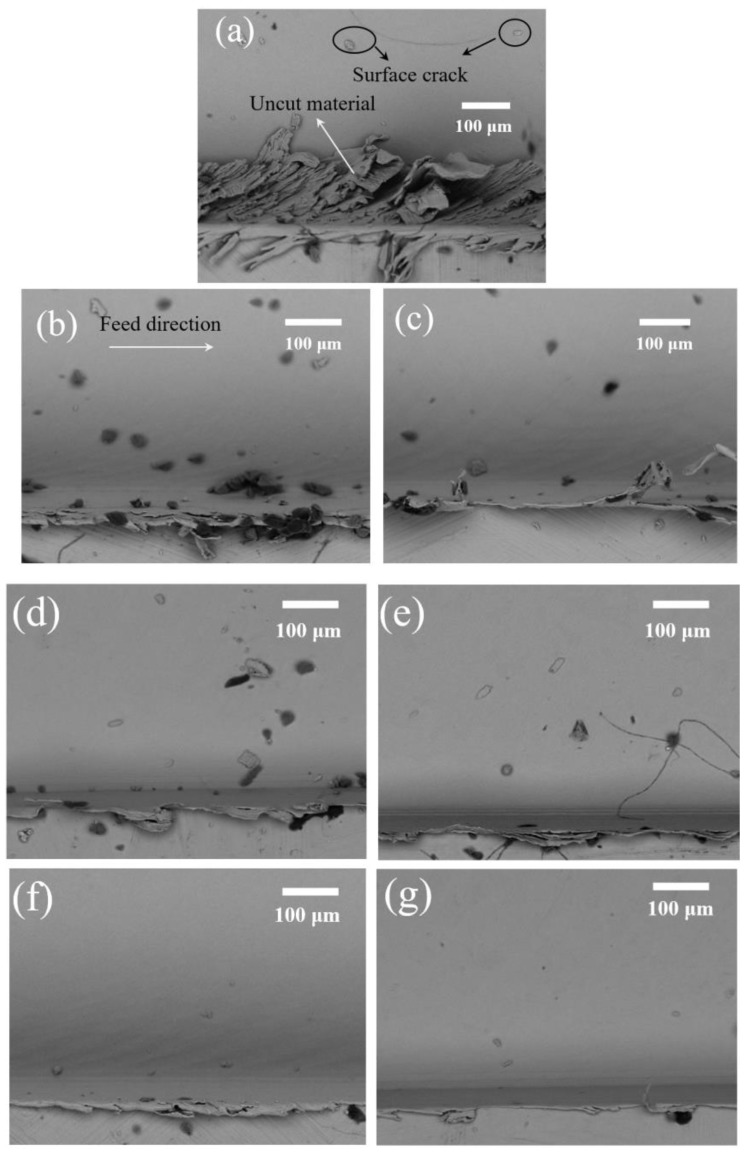
SEM measurement of burrs and the machined surface at the edge area of the up milling side of conventional micro milling and vibration-assisted micro milling at the cutting length of 600 mm. (**a**) Conventional micro milling; (**b**) a vibration amplitude of 0.5 µm and a vibration frequency of 1500 Hz; (**c**) a vibration amplitude of 1µm and a vibration frequency of 1500 Hz; (**d**) a vibration amplitude of 0.5 µm and a vibration frequency of 3500 Hz; (**e**) a vibration amplitude of 1 µm and a vibration frequency of 3500 Hz; (**f**) a vibration amplitude of 0.5 µm and a vibration frequency of 5500 Hz; (**g**) a vibration amplitude of 1 µm and a vibration frequency of 5500 Hz.

**Figure 15:**
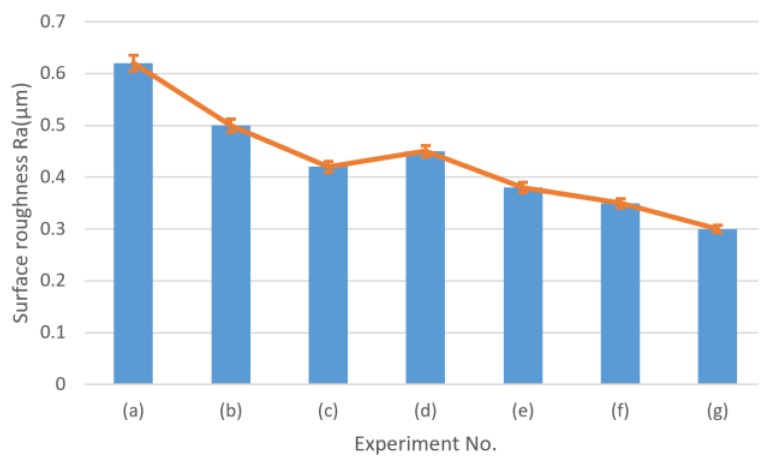
Surface roughness results corresponding to the SEM figures.

**Figure 16:**
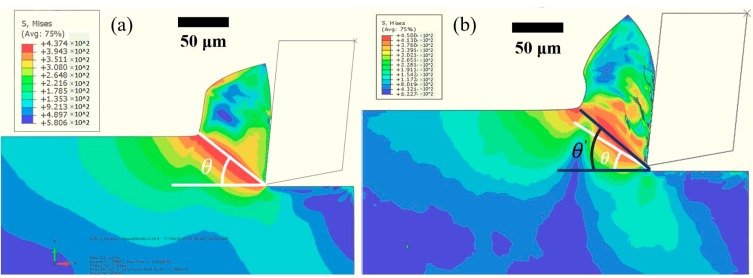
FE results of shear angle. (**a**) Conventional micro milling, (**b**) vibration-assisted micro milling with tool-workpiece separation.

Tool wear also has an important influence on the cutting performance, which includes surface finish and burr formation. It has been reported that the size effect will be amplified when a micro milling tool, especially a worn micro milling tool, cuts in and out of the workpiece. The amplified side effect exacerbates the ploughing effect at the edges of the machined slot and affects the surface finish and burr formation in that area [29]. Figure 14 shows the SEM measurement of burrs and the machined surface at the edge area of the up-milling side of conventional micro milling and vibration-assisted micro milling. Large top burr formation, worse surface finish, high surface roughness (Figure 15), surface cracks, and a large amount of uncut material can be observed on the machined result of conventional micro milling. Contrastingly, a smaller size of burrs, better surface finish, low surface roughness (Figure 15), and no uncut material can be found in the results of vibration-assisted micro milling. The best result of burr, the surface finish, and roughness can be observed at the vibration frequency of 5500 Hz and amplitude of 1 µm, which also agrees with the tool wear results. Generally, the burr size and surface crack and surface roughness will be decreased with the increase of vibration frequency and the condition of tool-workpiece separation. This mainly benefits from the low tool wear and the unique cutting mechanism. On one hand, the ploughing effect can be effectively reduced due to the lesser wear of the tool cutting edge radius and better tool geometry (smaller crater and less bonded workpiece materials on the tool). On the other hand, a bigger shear angle (Figure 16) can be obtained when tool-workpiece separation conditions are satisfied, leading to a lower uncut chip thickness and a smaller friction between the cutting tool and chips, improving the machining performance and further reducing tool wear [30]. As a result, better surface finish and surface roughness, smaller burr, and a lesser surface crack can be obtained by increasing vibration frequency and satisfying the tool-workpiece separation condition in vibration-assisted micro milling. 

## 6. Conclusions

This paper studied the tool wear suppression mechanisms in the vibration-assisted micro milling process. Machining experiments and finite element simulation are conducted by considering tool-workpiece separation conditions. The results show that the adhesive and mechanical wear of the micro tool can be effectively reduced as vibration is added, leading to an extended tool life and better cutting performance. The following conclusions can be drawn:

1. Tool wear can be reduced effectively as vibration is added and the minimized tool wear can be obtained as the tool-workpiece separation condition is satisfied due to the suppression of size and ploughing effect.
2. A coating layer loss initiates the wear mechanism for the coated tool, and adhesive and mechanical wear are the main wear mechanisms for the substrate wear of the cutting tools. These wear mechanisms can be suppressed effectively in vibration-assisted micro milling when tool-workpiece separation occurs, because the periodic separation between the cutting tool and workpiece enhances the cooling effect of the tool and reduces the cutting force as well as the generation of built-up edge.
3. The relationship between tool wear and cutting performance is studied. Compared with conventional micro milling, the cutting performance in vibration-assisted micro milling can be improved. The machining results also indicate that better surface finish, smaller burr, and lesser surface crack can be obtained by increasing the vibration frequency and satisfying the tool-workpiece separation condition due to the low tool wear and the unique cutting mechanism.

## Figures and Tables

**Table 1:** Machining and vibration parameters used in the experiments.

No	Vibration Amplitude (µm)	Vibration Frequency (Hz)	Spindle Speed (rpm)	Feed Rate (µm/tooth)	Axial Depth of Cut (µm)	Tool-Workpiece Separation
1	0	0	30,000	1.5	50	NO
2	0.5	1500	30,000	1.5	50	NO
3	1	1500	30,000	1.5	50	YES
4	0.5	3500	30,000	1.5	50	NO
5	1	3500	30,000	1.5	50	YES
6	0.5	5500	30,000	1.5	50	NO
7	1	5500	30,000	1.5	50	YES

**Table 2:** Mechanical properties and materials constant in the Johnson–Cook (J–C) model for titanium alloy [24].

Properties	Values
Density (ton/mm^3^)	4.5 × 10^−9^
Young’s Modulus (MPa)	96,832.3
Poisson’s Ratio	0.32
*A* (MPa)	1098
*B* (MPa)	1092
*n*	0.93
*m*	1.1
*C*	0.014
*d*_1_	−0.09
*d*_2_	0.25
*d*_3_	−0.5
*d*_4_	0.014
*d*_5_	3.87
